# One-Step Generation of Alginate-Based Hydrogel Foams Using CO_2_ for Simultaneous Foaming and Gelation

**DOI:** 10.3390/gels8070444

**Published:** 2022-07-16

**Authors:** Imene Ben Djemaa, Sébastien Andrieux, Stéphane Auguste, Leandro Jacomine, Malgorzata Tarnowska, Wiebke Drenckhan-Andreatta

**Affiliations:** 1 Institut Charles Sadron, CNRS UPR22-University of Strasbourg, 67084 Cedex 2 Strasbourg, France; imen.ben-djemaa@etu.unistra.fr (I.B.D.); sebastien.andrieux@ics-cnrs.unistra.fr (S.A.); jacomine@unistra.fr (L.J.); 2 Urgo Research Innovation and Development, 21300 Cedex Chenôve, France; s.auguste@fr.urgo.com (S.A.); m.tarnowska@fr.urgo.com (M.T.)

**Keywords:** hydrogel foams, liquid foam templating, alginate hydrogels, gas-initiated cross-linking, interfacial rheology

## Abstract

The reliable generation of hydrogel foams remains a challenge in a wide range of sectors, including food, cosmetic, agricultural, and medical applications. Using the example of calcium alginate foams, we introduce a novel foam generation method that uses CO_2_ for the simultaneous foaming and pH reduction of the alginate solution to trigger gelation. We show that gelled foams of different gas fractions can be generated in a simple one-step process. We macroscopically follow the acidification using a pH-responsive indicator and investigate the role of CO_2_ in foam ageing via foam stability measurements. Finally, we demonstrate the utility of interfacial rheology to provide evidence for the gelation process initiated by the dissolution of the CO_2_ from the dispersed phase. Both approaches, gas-initiated gelation and interfacial rheology for its characterization, can be readily transferred to other types of gases and formulations.

## 1. Introduction

Hydrogel foams are very attractive for biomedical [1,2,3,4,5], cosmetic [6], food [7], and agricultural applications [8] thanks to their absorption capacity, low density, large surface-to-volume ratio, biocompatibility, and mechanical properties. Since they are obtained by the gelling of an initially liquid foam via a method called “liquid foam templating” [9,10,11], a reliable foam generation process requires the fine-tuning of a wide range of interrelated timescales: foaming time, gelation time, and foam ageing time. For example, overly rapid gelation alters the rheological properties of the foaming liquid during foaming, leading to an inhomogeneous foam, while excessively slow gelation leads to foam collapse before solidification [11].

Special attention has been paid to alginate-based hydrogel foams on the basis of their biocompatibility and their interesting ion-bonding properties [8,12,13,14]. Most alginate hydrogels are physically cross-linked with multivalent-cations, the most popular being calcium-cross-linked alginates [15,16,17,18,19,20,21,22]. However, the external gelation of alginate solution by direct contact with calcium chloride tends to be too rapid to allow for the homogeneous mixing of calcium and alginate solutions, leading to hydrogels with structural heterogeneity and limited mechanical properties [23]. The external gelation of foamed alginate solutions imposes even more constraints on calcium diffusion, increasing the risk of gel heterogeneity [20]. To overcome this drawback, several studies have used microfluidics to generate alginate bubbles one by one and collect them in a calcium chloride solution [21,22]. However, microfluidics is limited by its low production rate [24]. A more efficient method for the cross-linking of alginates is internal cross-linking: using either the pH-dependent solubility of solid calcium-based salts (e.g., CaCO_3_) [17,25] or the pH-triggered release of calcium ions complexed with a chelating agent [26]. In both cases, gelation is initiated by the acidification of the alginate solution mixed with calcium in its inactive form. We shall call the solution of dissolved sodium alginate mixed with CaCO_3_ the “pre-gelling solution”. This method provides access to a wider range of foaming methods, including chemical foaming [15] and mechanical foaming [17,19,27,28,29,30]. Acidification is commonly ensured using glucono-delta-lactone (GDL), a compound which slowly hydrolyzes to release gluconic acid, leading to a slow decrease in pH, and, in turn, a slow and homogeneous release of calcium ions for cross-linking [23]. However, since GDL starts hydrolyzing as soon as it is dissolved, the kinetics of acidification, and therefore gelation, are closely linked with the kinetics of the dissolution and hydrolysis of GDL. Moreover, to generate hydrogel foams, the GDL needs to be added as a powder prior to foaming, which adds a strong constraint on the foaming time and limits the range of applicable foaming methods. An efficient technique for internal gelation applicable to a wide range of foaming methods is therefore still lacking.

**Figure 1:**
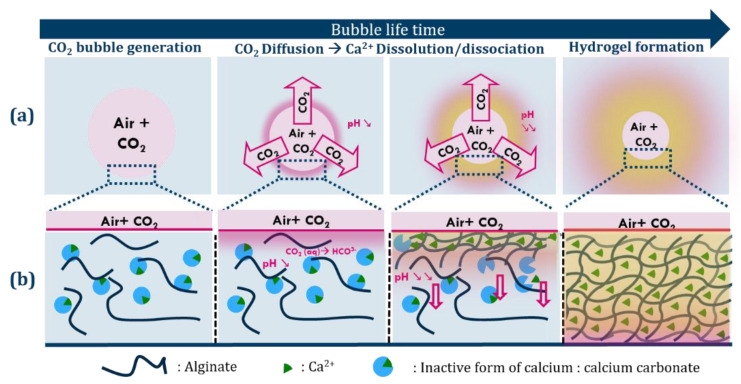
(**a**) Schematic representation of the internal cross-linking of alginate with inactive calcium ions (CaCO_3_) via acidification of the medium induced by the dissolution of CO_2_ from the bubbles. (**b**) Scheme of the diffusion of CO_2_ from the interface into the bulk and evolution of the gelling front with time. The growing hydrogel layer is represented in yellow.

To fill this gap, we introduce here an innovative approach which couples foaming and the triggering of gelation in a one-step process [31] by mechanical foaming with an air-CO_2_ mixture (Figure 1). The diffusion and dissolution of the CO_2_ from the foam bubbles into the alginate solution induces the interface-driven acidification of the initially basic pre-gelling solution via the following equilibria [32]:

CO_2(g)_ ⇋ CO_2(aq)_ (1)

CO_2(aq)_ + 2 H_2_O ⇌ H_2_CO_3_ + H_2_O ⇋ HCO_3_^−^ + H_3_O^+^ (2)

HCO_3_^−^ + H_2_O ⇌ CO_3_^2−^ + H_3_O^+^. (3)

This acidification induces the progressive release of the calcium ions around the bubbles and hence the homogeneous gelation of the overall foam. We show that the gelation process from the interface into the bulk of the continuous phase efficiently protects the foam from all relevant foam ageing mechanisms, leading to a stable hydrogel foam (Section 2.1). We furthermore introduce interfacial rheology as a convenient tool to monitor the gas-driven gelation process of an isolated interface (Section 2.2).

## 2. Results and Discussion

### 2.1. Foam-Scale Investigation of CO_2_ Effect on Alginate Foam Gelation

We produced foams with different gas fractions, *φ* (ratio of gas volume to foam volume), and CO_2_/air ratios using the double syringe technique (Section 4.2.1). The foaming solutions contained 1 wt% alginate and 0.2 M calcium carbonate microparticles (Section 4.1). In some cases, bromothymol blue was added as a pH indicator to observe the evolution of the pH following foaming. Since this dye reveals pH changes in the range between 6.2 (yellow) and 7.6 (blue) [33], the solution was blue (pH 10) before foaming.

**Figure 2:**
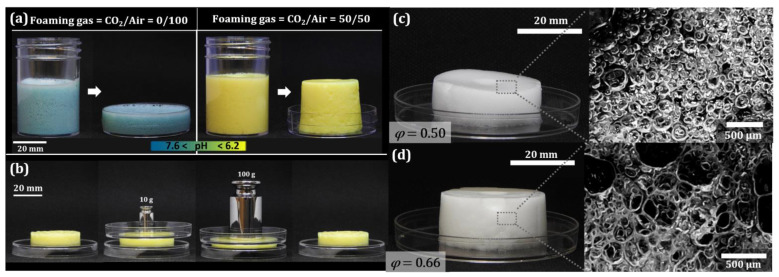
Foams obtained with different foaming gases: air (**a** (**left**)) vs. CO_2_/air = 50/50 (**a** (**right**),**b**–**d**). The foaming solution was a mixture of 1 wt% alginate, 0.02 M CaCO_3_, 2 wt% Disponil APG 425, and 5 × 10^−5^ M bromothymol blue as a pH-indicator dye. The morphology of the gelled foams could be controlled via the gas fraction *φ*: (**c**) *φ* = 0.5; (**d**) *φ* = 0.66 (both foams generated without pH indicator for better optical contrast).

Figure 2a (left) shows a foam generated with air as the foaming gas. The foam retained its initial blue color, i.e., the pH remained > 7.6 after foaming. Because the foam remained liquid, it could be poured from the container into a petri dish. However, a foam formed with a CO_2_/air volume ratio of 50/50 (Figure 2a, right) changed color from blue to yellow within a few seconds, revealing a rapid pH decrease down to ≤ 6.2. This pH corresponds to the pH of the maximum release of Ca^2+^ from CaCO_3_, as confirmed by ionometry (Section 6 in the supplementary materials). This foam retained the cylindrical shape of the vial after it was removed. When subjected to a force exerted by weights (Figure 2b), it demonstrated resistance to compression, confirmed by the fact that its shape returned to its initial form after the weight was removed (Figure 2b, right). The weight–deformation relation of this simple experiment allowed us to estimate that the Young’s modulus of the foam was about 1.4 kPa and that of the gel was about 13 kPa (Section 5 in the supplementary materials). Incorporating CO_2_ into the foaming gas thus led to the acidification and gelation of the foam.

The morphology of the obtained hydrogel foams could be controlled via the gas fraction *φ*. Figure 2c,d show two examples at a CO_2_/air volume ratio of 50/50: The foam in Figure 2c was generated with *φ* = 0.5, while the foam in Figure 2d was prepared with *φ* = 0.66. One can see from the microscope images that the bubbles in the foam with *φ* = 0.66 (Figure 2d) are deformed into polyhedral shapes [34,35], while the bubbles in the foam with *φ* = 0.5 remain fairly spherical and are not in contact with each other, indicating a morphology closer to a “bubbly hydrogel” than a foam.

To quantitatively investigate the effect of the addition of CO_2_ on the foam stability, we compared the ageing of a foam generated with air (CO_2_/air = 0/100) to that of a foam obtained with CO_2_/air = 50/50 (Section 4.2.1). 

**Figure 3:**
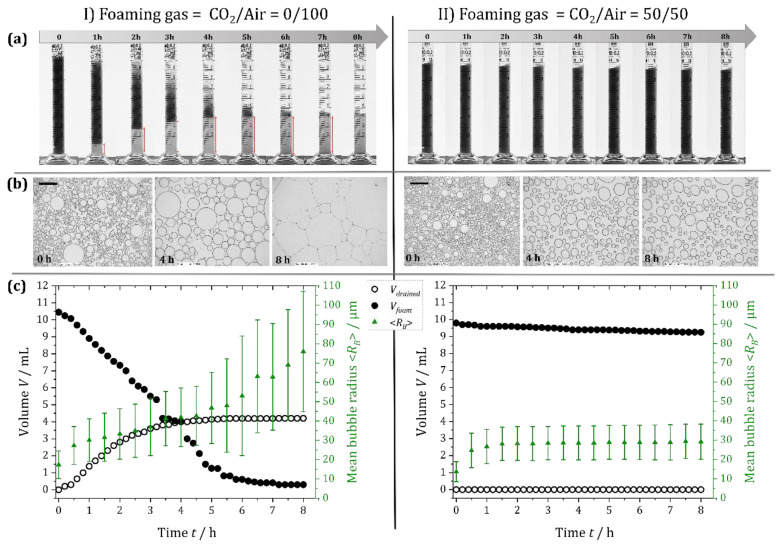
Foam stability of two alginate foams generated with (**I**) CO_2_/air = 0/100 and (**II**) CO_2_/air = 50/50. (**a**) Photographs of the foams taken in closed graduated glass cylinders at 1 h time intervals; the black zone corresponds to the foam, and the transparent zone at the bottom is the drained liquid. (**b**) Microscope images obtained over 8 h after squeezing the foam into a monolayer of bubbles between two glass plates separated by a 34 µm spacer (scale bars 500 µm). (**c**) Temporal evolution of the drained volume of liquid, *V*_drained_, and the foam volume, *V*_foam_, measured from (**a**), together with the mean bubble radius measured from (**b**). The error bars represent the standard deviation of the bubble sizes measured for each image.

The foam generated with CO_2_/air = 0/100 showed the classic features of an unstable foam [35]: an increasing volume of liquid, *V*_drained_, drained out of the foam under the action of gravity (transparent zone underneath the foam in Figure 3Ia and *V*_drained_ in Figure 3Ic), and the overall foam volume, *V*_foam_, decreased with time due to the coalescence of bubbles from the top of the foam (Figure 3Ia and *V*_foam_ in Figure 3Ic). Moreover, coalescence and gas exchange between bubbles led to a progressive increase in the mean bubble radius <*R*_B_> with time (green data in Figure 3Ic). The combination of all these mechanisms led to the progressive disappearance of the foam over the 8 h of observation.

We observed very different behavior for the foam generated with the CO_2_/air = 50/50 mixture. As shown in Figure 3IIa,c, the drainage of the liquid was completely stopped from the outset, which can be attributed to the rapid gelation of the continuous phase. Moreover, the foam height remained constant, indicating the absence of bubble coalescence from the top of the foam. Nevertheless, the average bubble radius <*R*_B_> evolved over the first hour due to the CO_2_ dissolution and gas exchange between bubbles driven by the difference in the Laplace pressure between bubbles of different sizes. This evolution stopped when the liquid is saturated with CO_2_ and when the elastic modulus of the continuous phase was high enough to counteract the driving pressure difference [36].

While the reported observations clearly prove the gelation of the foam thanks to the CO_2_, future investigations should establish more quantitatively the influence of the CO_2_ content, gas fraction *φ*, bubble size *R*_B_, and alginate concentration on the gelation kinetics and associated foam stability, as has been achieved in the past for gelatin foams [36].

### 2.2. Interfacial Investigation of CO_2_ Effect on Alginate Gelation

**Figure 4:**
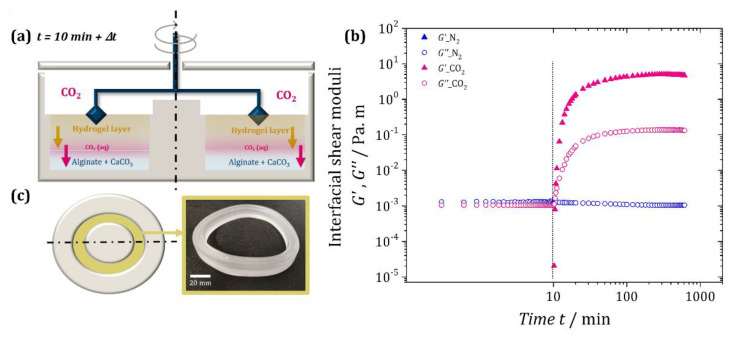
(**a**) Scheme of the double-wall ring (DWR) geometry used for the interfacial shear rheology, showing our understanding of what happens in the presence of CO_2_: The blue layer at the bottom represents the un-gelled solution (1 wt% alginate, 0.02 M CaCO_3_). The progressing front of dissolved CO_2_ (i.e., acidification) is shown in pink, and the progressing gelation front on top in yellow. (**b**) Temporal evolution of the interfacial elastic shear modulus, *G*′, and loss modulus, *G*″, of the gas/liquid surface of the alginate solution when a gas flow of N_2_ (blue) or CO_2_ (pink) is switched on after 10 min of exposure to ambient air (vertical line). (**c**) Hydrogel sample extracted after the experiment using CO_2_ in the form of a ring maintaining the shape of the geometry.

To obtain a more quantitative characterization of the interface-driven CO_2_-induced gelation, we characterized the interfacial properties of the alginate solution mixed with calcium carbonate microparticles using interfacial shear rheology in the absence and presence of CO_2_ (Section 4.2.2). The double-wall ring geometry [37] of the setup is sketched in Figure 4a.

Figure 4b shows the evolution of the interfacial elastic and loss modulus, *G*′ and *G*″, respectively, over time for the two experiments. During the first 10 min under ambient air, the interfacial elastic modulus *G*′ was below the measurement threshold, while the interfacial loss modulus *G*″ was about 10^−3^ Pa m. This is characteristic of purely viscous interfaces [38,39]. Most importantly, the measured values remained constant over the 10 min under an airflow, indicating the absence of gelation. After switching on the N_2_ flow (blue curve after the vertical line, Figure 4b), *G*′ remained below the detection limit and the interfacial loss modulus *G*″ also remained nearly constant. Therefore, the interface remained in a purely viscous state, indicating that the N_2_ flow did not trigger gelation. This was very different when the CO_2_ flow was switched on (pink curve after vertical line, Figure 4b). Both the interfacial elastic and loss modulus increased rapidly, with the elastic modulus *G*′ exceeding the loss modulus *G*″ after less than a minute (*G*′ > *G*″). This indicated a sol–gel transition [39,40,41] caused by the progressive gelation of the alginate starting from the liquid/gas interface, as sketched in Figure 4a. As the gelling front progressed into the solution upon the dissolution/diffusion of CO_2_, both *G*′ and *G*″ kept increasing until a plateau was reached after ca. 100 min. Since alginate hydrogels are reported in the literature to have mesh sizes in the range of 7–20 nm [42,43,44], much larger than the diameter of a CO_2_ molecule (0.33 nm), we may consider that the diffusion/dissolution of CO_2_ remained unhindered by the hydrogel formation. Hypothesizing a diffusion-limited process with a diffusion constant of *D* = 1.89 × 10^−9^ m^2^/s (at 1 atm and 25 °C) for CO_2_ in water [45] (which we confirm in Section 1 in the supplementary materials), we can then estimate that at *t* = 100 min, the CO_2_ had penetrated roughly *L* = (6*Dt)*^1/2^ ≈ 8 mm into the liquid. This means that it is likely that the acidification and the gelation front had reached the bottom of the trough (10 mm), which may explain the plateau. This hypothesis is also supported by the fact that the gel at the end of the experiment could be removed in one solid piece from the trough, as shown in Figure 4c. The obtained bulk hydrogel suggests that CO_2_ introduction via gas flow above the solution may also be an interesting method for formulating bulk hydrogels using CO_2_ instead of other acids.

We can use the same estimations as above to approximate the characteristic gelation time of the foams (see Figure S1). Considering a foam with an average bubble radius of <*R*_B_> = 100 µm and nearly spherical bubbles, we can estimate that the liquid zones between the bubbles have approximately the same characteristic thickness [35]. Hence, it will take about *t* = (100 µm)^2^/(6D) ≈ 1 s for the CO_2_ to diffuse into the continuous phase of the foam. This calculation also agrees with the interfacial rheology experiment (Figure 4b), in which the viscoelastic moduli of the interface evolved dramatically over the first few seconds of CO_2_ flow. Interfacial rheology therefore constitutes a promising method for the characterization of interface-induced gelation and could easily be applied to other systems with interface-triggered solidification. However, a truly quantitative interpretation of the viscoelastic moduli requires appropriate modelling of the gelation process and the modification of the analysis software of the rheometer, since the current tool was developed for nano- to micrometer-thick interfacial layers in contact with a homogeneous liquid of constant viscosity. In our gelation experiments, this holds true only for the very first instances. Hence, the quantitative values from more than a few seconds after switching on the CO_2_ flow should only be taken as indicators.

## 3. Conclusions

In summary, we reported for the first time the generation of alginate-based hydrogel foams using the foaming gas as the gelation trigger in a simple one-step process. We showed that foaming with a gas mixture containing CO_2_ allowed the acidification and thus the gelation of the continuous phase and efficiently stopped all the main mechanisms of foam ageing. The different timescales of the foaming/gelation process and the final foam properties could be adjusted via the CO_2_ fraction of the foaming gas and the gas fraction (*φ*) of the initially liquid foam. Here, we focused on a general introduction to the method to show its feasibility. Different physico-chemical and physical parameters are involved in this system whose quantitative influence remains to be established via a fully systematic approach. The physico-chemical parameters include the structural properties of the sodium alginate (its molecular weight and its mannuronic-acid-to-guluronic-acid ratio); its concentration; the concentration of CaCO_3_; the choice of the surfactant; the CO_2_/air ratio; and the use of additives. The physical parameters include the bubble size and the gas fraction. These parameters are largely interconnected, requiring in-depth investigation, ideally coupled with an analysis of the final foam in terms of its mechanical properties.

We also showed that the characteristic timescales and evolution of the mechanical properties of the interface-driven gelation can be demonstrated using interfacial shear rheology—even though a fully quantitative interpretation of the obtained values requires the adaptation of the analysis algorithms.

It needs to be kept in mind that for the gelled foams to remain stable, they have to be contained in a sealed environment, since otherwise, the CO_2_ will slowly leave the foam. This will lead to a progressive increase in the pH and hence a progressive “degelling” of the foam matrix, which will render the foam sensitive again to the different ageing mechanisms, leading to its final destruction. The duration of this gelation–degelation cycle can be controlled by the initial CO_2_ content, gas fraction (*φ*), bubble size (*R*_B_), and alginate type/concentration. It may therefore be of interest for processes wherein a highly stable (gelled) foam is only needed for a specific period of time.

Last but not least, both foaming and interfacial rheology can easily be extended to other pH-sensitive polymers or proteins [46,47,48,49,50] (and potentially to other water-soluble gas components). We therefore believe that our findings may inspire much wider activities pertaining to gas-initiated material fabrication.

## 4. Materials and Methods

### 4.1. Materials

We used a pre-gelling solution of 1 wt% alginic acid sodium salt (purchased from Alfa Aesar), which was solubilized in Milli-Q water under mechanical stirring at 50 °C for 3 h. We dispersed 0.02 M of calcium carbonate microparticles with an average size of 5 µm (purchased from Alfa Aesar) in the alginate solution by magnetic mixing for 30 min together with 2 wt% of the surfactant Disponil APG 425 (an alkyl polyglycoside, from BASF). The initial pH of this solution was 10. All solutions were prepared in contact with air and were therefore considered to be in equilibrium with the natural CO_2_ content of air (~0.04%) before and after foaming with air.

In some cases, 5 × 10^−5^ M of bromothymol blue was added to the solution (with calcium bicarbonate salt) as the pH-sensitive indicator. This dye reveals pH changes in the range between 6.2 (yellow) and 7.6 (blue) [33]. The foaming solution was therefore blue before foaming.

### 4.2. Methods

#### 4.2.1. Foaming and Foam Ageing Analysis

We produced the foams using the double-syringe technique [51](see Figure S2), which consists of mixing the foaming solution with the selected gas mixture by repeatedly pushing the gas/liquid mixture from one syringe to another (4 cycles) for ca. 10 s. The overall gas fraction *φ* (gas volume/foam volume) of the initially liquid foam was controlled by how much gas was added to the syringes with respect to the liquid volume. The final gas fraction of the hydrogel foam was slightly smaller, since some of the CO_2_ dissolved in the liquid. Gas mixtures with particular CO_2_/air volume ratios were prepared by filling the syringe first with a given volume of CO_2_, collected above dry ice, to which a given volume of air was added at ambient pressure.

The generated foams were collected in graduated cylinders of 12 mm internal diameter, and their evolution over time was captured using a digital camera over eight hours following their generation. In parallel, we squeezed part of the foam between two sealed glass plates separated by 34 µm to create a monolayer of bubbles (see Figure S3) for the easier characterization of the evolution of the mean bubble radius <*R*_B_>.

#### 4.2.2. Interfacial Shear Rheology

The interfacial shear rheology experiments were conducted using a DHR-3 rheometer (TA instruments) with a double-wall ring (DWR) geometry [37,52] and the same pre-gelling solutions as were used for the foaming experiments, though without surfactant to avoid interferences related to the viscoelastic properties of the surfactant monolayer.

To obtain the viscoelastic moduli of the interface, the ring was oscillated at a frequency of 0.2 Hz and a strain amplitude of 1%. After 10 min of measurement in ambient air, a controlled gas flow was generated above the liquid using either N_2_ from a compressed gas bottle or CO_2_ collected above dry ice in an ice-bathed steel bottle. The gas passed through a flowmeter set at 150 mL/min and was humidified by bubbling through water previously saturated with N_2_ or CO_2_, respectively (see Figure S4). This humidification was important to prevent the drying of the liquid surface due to the gas flows, which could be misinterpreted as gelation.

## Supplementary Materials

The following supporting information can be downloaded at: https://www.mdpi.com/article/10.3390/gels8070444/s1, Figure S1. (a) The evolution of the diffusion length (L) of aqueous CO_2_ dissolved from a bubble of radius 2.2 mm. The line is the theoretical prediction using the well-known diffusion constant of D = 1.89 × 10^−^^9^ m2/s for CO_2_ in water (at 1 atm and 25° C). (b) Selected images of the bubble (black circle and the surrounding ring) with the propagating CO_2_ front (dark region) taken at t = 0 s, t = 20 s, t = 40 s, and t = 60 s; Figure S2. Setup for double-syringe foaming technique; Figure S3. Left—Setup for obtaining a monolayer of foam. Right—Steps for image treatment using ImageJ: (a) the original image recorded by microscope, (b) conversion to binary image (8 bits), (c) thresholding, (d) automatic detection of circular objects and area measurement [51]; Figure S4. Top—Setups for introducing (a) CO_2_ or (b) N_2_ flow into the rheometer. The central picture shows a zoom-in on the double-wall ring (DWR) geometry used for this experiment, in which the sample was loaded. Bottom—Schematic drawings of the corresponding setups; Figure S5. Left: Simple compression tests on a hydrogel foam obtained by foaming a mixture of 1 wt% alginate, 0.02 M CaCO_3_, 2 wt% Disponil APG 425, and 5 × 10^−5^ M bromothymol with CO_2_/air = 50/50. Right: Obtained values of the applied stresses as a function of the resulting de-formation with a linear fit [36,53]; Figure S6. Amplitude sweep test on a hydrogel (1 wt% alginate, 0.02 M CaCO_3_, 1 wt%) gelled using GDL instead of CO_2_. Figure S7. Percentage of released calcium ions from CaCO_3_ as a function of pH.
