# Supplementary material for: One-Step Generation of Alginate-Based Hydrogel Foams Using CO2 for Simultaneous Foaming and Gelation
Source: Gels. 2022 Jul 16;8(7):444. doi: 10.3390/gels8070444 (PMC9322084; doi:10.3390/gels8070444)
Supplement: Supplementary file 1 [file gels-08-00444-s001.zip › gels-1751387-supplementary.pdf]

gels

MDPI

Communication

One-Step Generation of Alginate-Based Hydrogel Foams Using CO_2 for Simultaneous Foaming and Gelation

Imene Ben Djemaa [1,2], Sébastien Andrieux [1], Stéphane Auguste [2], Leandro Jacomine [1], Malgorzata Tarnowska [2] and Wiebke Drenckhan-Andreatta [1,*]

[1] Institut Charles Sadron, CNRS UPR22-University of Strasbourg, CEDEX 2, 67084 Strasbourg, France; imen.ben-djemaa@etu.unistra.fr (I.B.D.); sebastien.andrieux@ics-cnrs.unistra.fr (S.A.); jacomine@unistra.fr (L.J.)

[2] Urgo Research Innovation and Development, CEDEX, 21300 Chenôve, France; s.auguste@fr.urgo.com (S.A.); m.tarnowska@fr.urgo.com (M.T.)

* Correspondence: drenckhan@unistra.fr

Supplementary Materials

Citation: Ben Djemaa, I.; Andrieux, S.; Auguste, S.; Jacomine, L.; Tarnowska, M.; Drenckhan-Andreatta, W. One-Step Generation of Alginate-Based Hydrogel Foams Using CO_2 for Simultaneous Foaming and Gelation. *Gels* **2022**, *8*, 444. https://doi.org/10.3390/gels8070444

Academic Editors: Hongjie Dai, Zhili Wan and Juntao Tang

Received: 16 May 2022
Accepted: 22 June 2022
Published: 16 July 2022

Publisher's Note: MDPI stays neutral with regard to jurisdictional claims in published maps and institutional affiliations.

Copyright: © 2022 by the authors. Licensee MDPI, Basel, Switzerland. This article is an open access article distributed under the terms and conditions of the Creative Commons Attribution (CC BY) license (https://creativecommons.org/licenses/by/4.0/).

1. Evolution of acidification front from a single CO_2 bubble

To follow the diffusion-driven transport of aqueous CO_2 dissolved from a single CO_2 bubble, we used a pH-sensitive dye (sodium fluorescein from Sigma Aldrich at 2×10^{-4} M) and a fluorescent microscope from Dino-lite (AM4115T-GFBW). The bubble was confined between two plates separated by a rubber O-ring of 1.6 mm thickness. We generated a bubble of 2.2 mm radius and observed the radial trajectory of the acidification front (Figure S1). The diffusion length (L) of the aqueous CO_2 dissolved from the bubble was calculated by subtracting the radius of the dark region surrounding the bubble (i.e., the acidified volume of the surrounding solution) from the radius of the bubble, considering both areas as disks. We measured L every 5 s for 1 min and compared its evolution over time with the characteristic diffusion time of CO_2 in water, considering a diffusion constant of $D = 1.89 \times 10^{-9}$ m²/s (at 1 atm and 25° C) for CO_2 in water. The calculated L (L_{theo}) and the experimentally measured L correlated well, which suggests that the estimations we made could be used to approximate the characteristic acidification time (i.e., gelation time) of the foam.

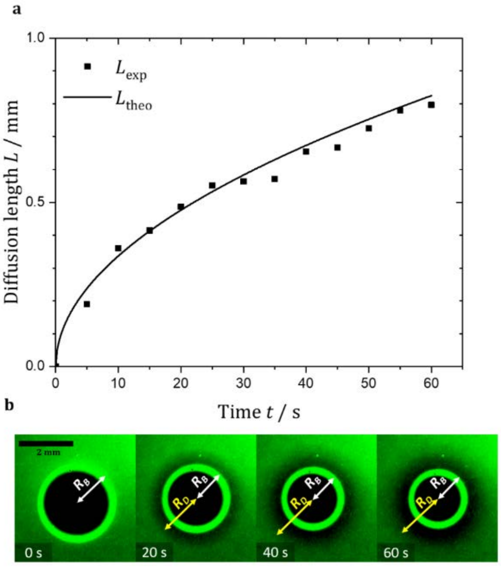

Figure S1. (**a**) The evolution of the diffusion length (L) of aqueous CO_2 dissolved from a bubble of radius 2.2 mm. The line is the theoretical prediction using the well-known diffusion constant of $D = 1.89 \times 10^{-9}$

m^2/s for CO_2 in water (at 1 atm and 25° C). (**b**) Selected images of the bubble (black circle and the surrounding ring) with the propagating CO_2 front (dark region) taken at t = 0 s, t = 20 s, t = 40 s, and t = 60 s.

2. Experimental setup for foam generation

We used the double-syringe technique for foam generation in all presented experiments (Figures 2 and 3). The setup consisted of two identical plastic syringes of 60 mL connected with a constriction, as shown in Figure S2. One syringe was filled with the foaming liquid, the other with the selected gas. By repeatedly pushing between the two syringes through the constriction, we mixed the gas with the solution and hence caused the formation of the foam after 10 cycles.

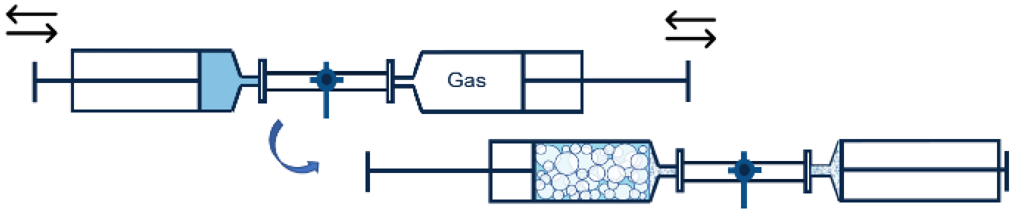

Figure S2. Setup for double-syringe foaming technique.

3. Bubble size measurements

Figure 3 shows the measurements of the bubble size of the produced foams over time. The microscopic observations were conducted using transmitted light microscopy (Keyence VHX 2000, Chicago, IL, USA). Foam samples were extracted directly from the output of the syringe immediately after foam generation and were placed between two microscope slides that were separated by an adhesive tape of known thickness (34 μm), taking on the role of a spacer. This spacing was chosen to obtain a monolayer of bubbles to facilitate bubble size measurement.

The automatically recorded images were analyzed using ImageJ. First, we set the scale according to the scale bar of the microscopic image. Then, the image was binarized and thresholded as shown in Figure S3. We then selected the smallest bubble we could spot in the image and measured its area. This area was fixed as the minimal surface of the bubbles that could be detected by the automatic bubble size measurement tool provided by the software. Once we obtained data from the detected bubbles, we searched for undetected bubbles and measured their areas manually. Finally, using the line tool, we measured the thickness of the bubble outlines (δ), which was necessary for calculating the radii of the bubbles.

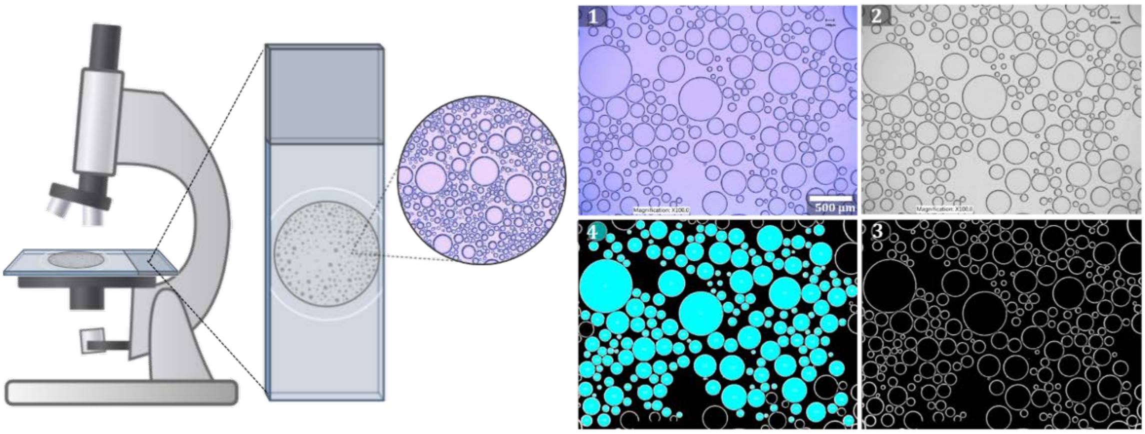

Figure S3. Left—Setup for obtaining a monolayer of foam. Right—Steps for image treatment using ImageJ: (**1**) the original image recorded by microscope, (**2**) conversion to binary image (8 bits), (**3**) thresholding, (**4**) automatic detection of circular objects and area measurement.

ImageJ allowed the calculation of the areas of circular objects, including spherical bubbles or squeezed bubbles in the form of a pancake. We used these data to convert the areas to the real radii of the bubbles following a method introduced by Gaillard et al. [51]. Finally, we determined the average bubble size <R_b> of each image and the standard deviation of all detected bubbles.

4. Experimental setup for interfacial rheology tests

Interfacial rheology experiments were conducted using a DHR-3 rheometer (TA instruments) with a double-wall ring (DWR) geometry. The setup for the experiments using N_2 or CO_2 as the gas phase are shown in Figure S4. We used compressed N_2 or dry ice (CO_2) contained in an ice-bathed steel bottle as the gas sources. The gas passed through a flowmeter set at 150 mL/min (this flow rate ensured the total exchange of the air previously filling the geometry chamber with the selected gas) and was then humidified (to prevent any drying that could be misinterpreted as gelation) by bubbling in water previously saturated with N_2 or CO_2. Finally, the gas inlets were connected to the rheometer.

Before starting each measurement, we proceeded with calibrating the geometry and sample conditioning at 25 °C. The geometry was covered with a solvent trap to avoid evaporation and limit contact with the atmosphere. Time-sweep tests were conducted at a frequency of 0.2 Hz and an amplitude of 1%. We first measured the initial viscoelastic moduli of the interface before gas introduction for 10 min and then introduced the gas flow and observed their variation.

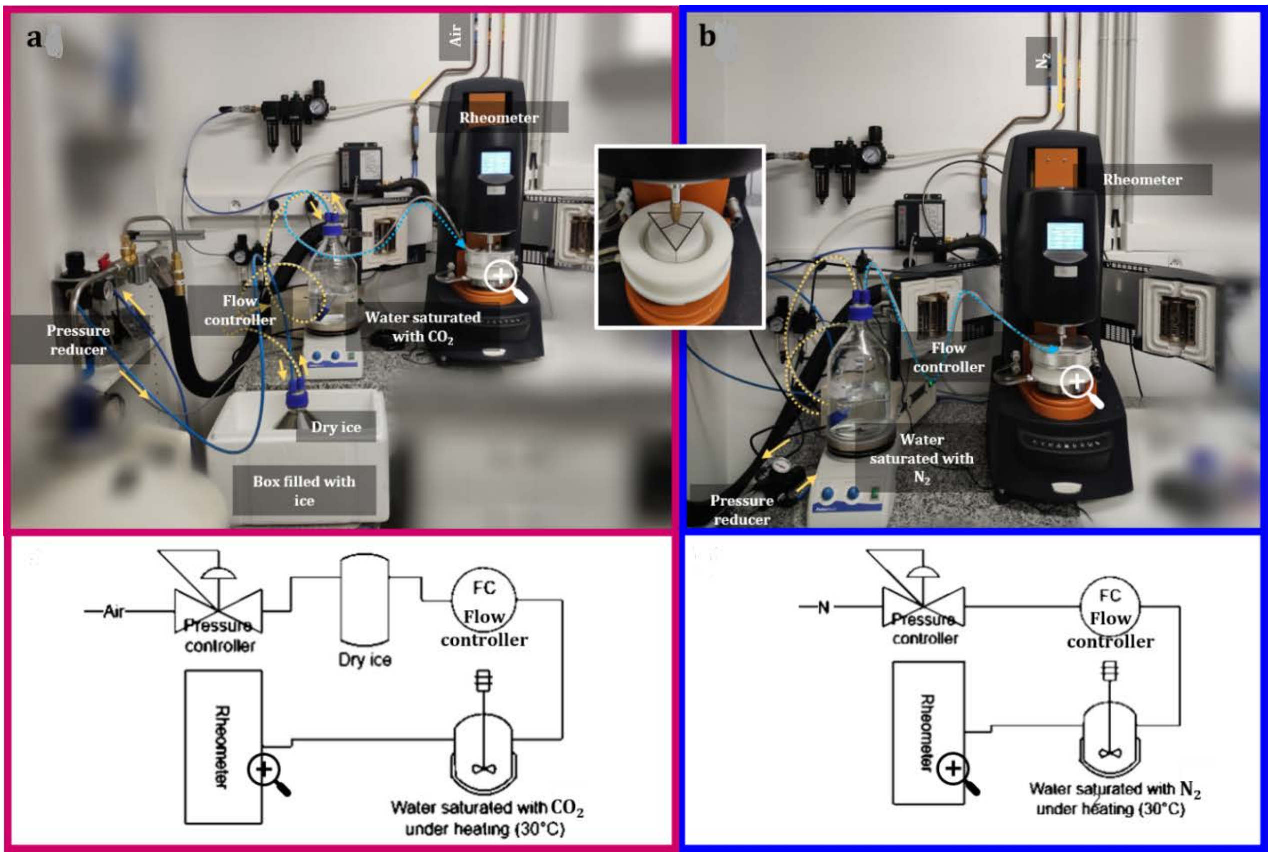

Figure S4. Top—Setups for introducing (**a**) CO_2 or (**b**) N_2 flow into the rheometer. The central picture shows a zoom-in on the double-wall ring (DWR) geometry used for this experiment, in which the sample was loaded. Bottom—Schematic drawings of the corresponding setups.

5. Estimation of Young's modulus of hydrogel foam

To obtain an estimation of the Young's modulus E of the foam, we used different calibration weights of known mass and observed the deformation of the hydrogel foam (left of Figure S5). The resulting stress–strain curve is shown on the right of Figure S5.

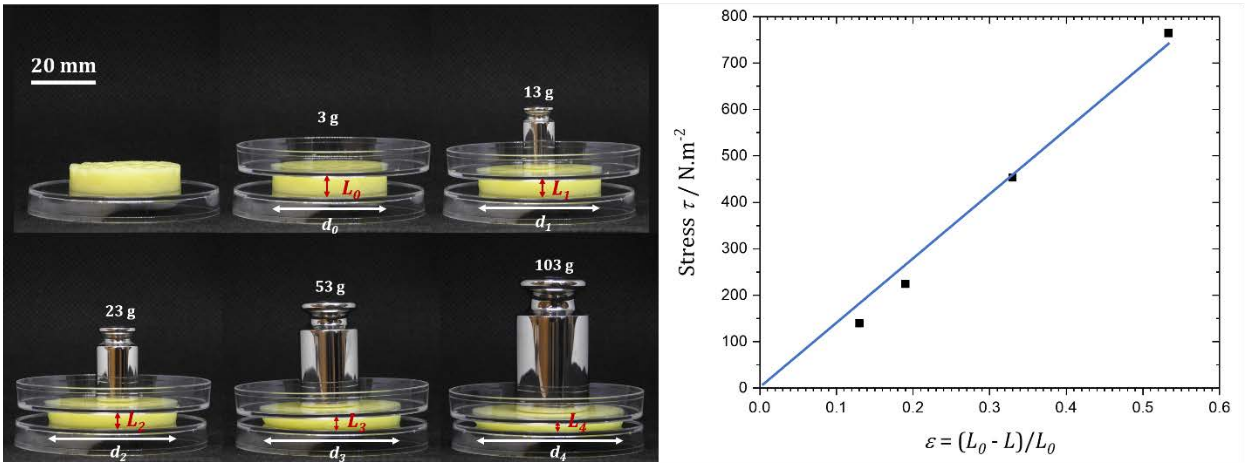

Figure S5. Left: Simple compression tests on a hydrogel foam obtained by foaming a mixture of 1 wt% alginate, 0.02 M $CaCO_3$, 2 wt% Disponil APG 425, and 5×10^{-5} M bromothymol with CO_2/air = 50/50. Right: Obtained values of the applied stresses as a function of the resulting deformation with a linear fit.

A linear fit through these data (passing through zero) gives

$\tau = (1386\ \mathrm{Nm}^{-2})\ \varepsilon$

As the slope of this line represents the Young's modulus E_{foam}, we can therefore estimate that $E_{foam} \approx 1.4$ kPa.

Since the gas fraction of this foam is 0.66, we can estimate the elastic modulus of the bulk gel to be [52,53]:

$E_{bulk} \approx E_{foam}\,(1\text{-}\varphi)^{-2} \approx 12.6$ kPa.

This may be compared to the bulk modulus of a hydrogel obtained from the same formulation using a different gelation trigger (glucono-delta-lactone (GDL) instead of CO_2). Figure S6 shows a typical amplitude sweep test on such a bulk alginate hydrogel. The test was preceded by a conditioning step at 23°C for 4 min with a 60 mm parallel plate (Peltier plate steel) at a gap size fixed by applying a small axial compression force of 1 N. The frequency was set at 0.1 Hz, and the strain ranged from 0.01 to 50 %. From this, we determine the shear modulus in the low deformation limit as G′ ≈ 3.4 kPa. Assuming an incompressible gel, this gives $E_{bulk,GDL} \approx 3$ G′ = 10.2 kPa. This value compares well to that obtained for the CO_2-based foam, considering the different approximations made and the difference in gelation method.

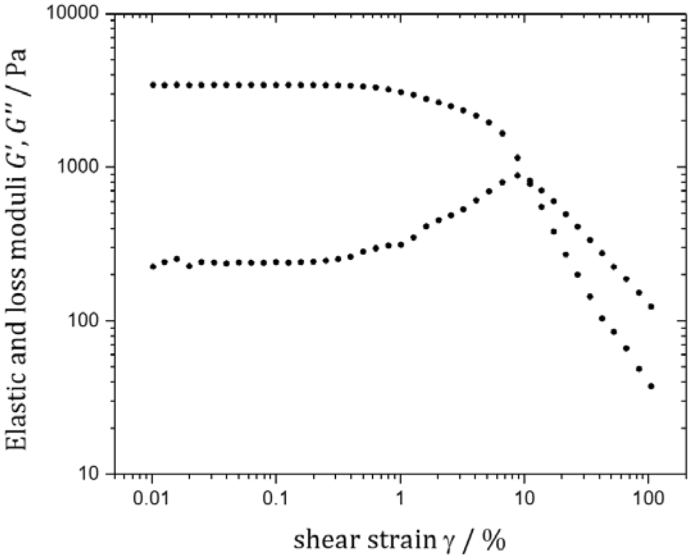

Figure S6. Amplitude sweep test on a hydrogel (1 wt % alginate, 0.02 M $CaCO_3$, 1 wt %) gelled using GDL instead of CO_2.

6. Ionometry experiment on $CaCO_3$ dissolution with acidification

An ionometry/pH-metry coupled experiment was conducted with a multi-channel instrument from SevenExcellence, using an InLab NMR sensor from Mettler for pH measurement and a calcium-selective electrode with an integrated reference for the determination of free calcium ion concentrations. The electrodes were connected to the multi-channel instrument, which allowed the simultaneous measurement of the two parameters for the same sample. Coding the LabX software allowed the autoEfram and simultaneous acquisition of data as a function of time. We show in Figure S7 the results obtained for a solution of 0.02 M $CaCO_3$, to which we added 1 wt % GDL when running the experiment.

Figure S7 shows that the calcium release was nearly insignificant at a pH range between 10 and 8.4, before it increased with acidification until reaching a maximum at a pH around 6.3, which corresponds to 95% of the calcium released from the initially dispersed $CaCO_3$.

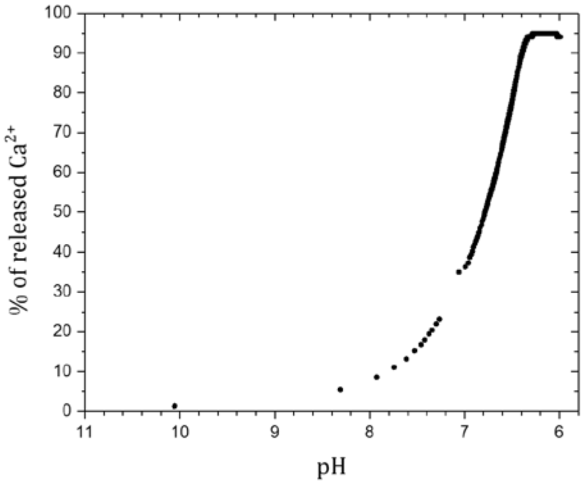

Figure S7. Percentage of released calcium ions from $CaCO_3$ as a function of pH.
